# Oxygen as obturation biomaterial in endodontic treatment: development of novel membranous dental restoration system

**DOI:** 10.12688/f1000research.132479.2

**Published:** 2024-11-06

**Authors:** Didi Wahyudi, Citra Kusumasari

**Affiliations:** 1 Dental Cooperation Indonesia, Bandung, 40134, Indonesia; 2 Department of Conservative Dentistry, Faculty of Dentistry, Universitas Indonesia, Depok, West Java, 16424, Indonesia

**Keywords:** dental restoration system, Enterococcus faecalis, obturation biomaterials, oxygen permeable membrane, endodontics treatments; trepanation

## Abstract

Complexities in obturation and difficulties in disinfection are the major problems that make endodontic treatment very time-consuming. A new perspective is needed to reduce the working time as well as to answer these two problems. Until now, none of the established obturation techniques for root canal filling can guarantee a perfect seal. Solid substances cannot be manipulated easily to fill the tiny branches of the root canal system hermetically. At the same time, anaerobes and facultative anaerobes, especially *Enterococcus faecalis*, are very dominant in endodontic infections discussion. As shown in many studies, it is extremely difficult to perfectly disinfect *Enterococcus faecalis* even by using some irrigating solutions with strong antibacterial properties. Under anaerobic conditions, the invasion efficiency of facultative anaerobes is increased. In case irrigation and disinfection cannot totally eliminate anaerobes and facultative anaerobes, a new strategy is also needed to deal with the bacteria that still survive in the root canal. Oxygen can fill the root canal system with ease, eliminate anaerobes, and prevent facultative anaerobes from being pathogenic. Therefore, using oxygen as obturation biomaterial after proper cleaning and shaping procedures is expected to solve the two major endodontic problems. The aim of this article was to discuss a new possible concept of dental restoration system that uses an oxygen-permeable membrane to decrease the time required in endodontic treatment. The membrane is placed at the orifice of a duct created to connect the entire root canal system with free air outside the restoration. The function of the membrane is more or less similar to the mask used during the COVID-19 pandemic, as it enables the oxygen to circulate while preventing any fluid, debris, and microorganisms from passing. We hypothesize that the oxygen circulating in the root canal system will also act as an antimicrobial agent that is constantly renewed.

## Introduction

As of March 2023, some variants of concern of severe acute respiratory syndrome coronavirus 2 (SARS-CoV-2) or coronavirus disease 2019 (COVID-19) hav been recorded. ^1^ Dentists are very vulnerable to being infected by COVID-19 because their face when treating patients is relatively close to the patient’s mouth. Saliva can be a place for various viruses to reside, including coronavirus, ^2^ and is something that dentists cannot wholly avoid when performing any procedure in the oral cavity. Therefore, dentists and patients must be careful when providing or having dental care.

Various ways have been implemented to deal with the spread of COVID-19 in dental practice, from shutting down dental clinics to limiting certain types of treatment that can only be administered using strict protocols. ^3^ The reason for all those decisions is to avoid or shorten interactions with patients who may possibly transmit the coronavirus. On the other hand, patients try not to visit places they consider potentially contaminated with coronavirus, such as hospitals and dental clinics. Hitherto, the COVID-19 pandemic has made a big impact to the way dentists work.

Endodontic treatment tends to require multiple visits, which can be a disadvantage for patients. However, root canal treatment may still be necessary for those experiencing severe dental pain. Most emergency cases in dentistry require endodontic treatment. ^4^ In a study conducted in India during the COVID-19 pandemic, there were three main reasons for emergency visits to dental clinics: pulpal problems 46.0%, abscess 16.6%, periapical lesions 15%, and most of the dental emergencies were endodontic related. ^5^

## Current endodontic treatment

There are ten principles that we can consider in endodontic treatment. The first and second principles are aseptic technique and the instruments that should be confined to the root canal. Third, root canal preparation should be performed by using proper instruments. Fourth, the root canal should be expanded, if possible, to make cleaning easier. Fifth, the root canal should be thoroughly irrigated with antiseptic solutions. Sixth, any use of irrigating solutions must be safe for periapical tissues. Seventh, if there is a sinus tract, it should be cleared up following root canal therapy and does not require surgery. An incision of the soft tissue can be performed for the acute periapical abscess to provide drainage. Eighth, the root canal should be hermetically obturated to give a good seal. Ninth, before obturation, a negative culture should be taken. Tenth, everything that is used in obturation should be biocompatible. These all are based on what was concluded in the International Conference on Endodontics in 1958. ^6^^–^^8^

There are three factors in endodontic treatment that must be considered for successful root canal therapy, namely, cleaning and shaping, disinfection, and obturation. ^9^ Periradicular pathosis is primarily caused by the growth of pathogens in the root canal system. Unsuccessful root canal therapy is not directly caused by the errors in performing endodontic treatment. ^10^

### Cleaning and shaping

Cleaning and shaping are separate and distinct concepts but performed concurrently. Some most significant factors influencing the result of cleaning process are the anatomy and morphology of the tooth, as well as the instruments and irrigants available during the process. The main objective of shaping is to maintain or develop a continuously tapering funnel from orifice to apex. Irritants should be reduced as much as possible, if not entirely. An adequately prepared root canal should feel smooth in all dimensions when the tip of a small file is pushed against the canal wall. After cleaning and shaping, sufficient space should be available for placing obturation materials. Irrigation with 17% ethylenediaminetetraacetic (EDTA) for 1 minute is recommended to remove the smear layer accumulated on the radicular canal wall. ^11^ The success of root canal therapy is majorly achieved by proper cleaning and shaping. Endodontically treated teeth fail not because of poor obturation but due to poor cleaning and shaping. ^9^^,^^12^

### Disinfection

Most clinicians choose sodium hypochlorite (NaOCl) as it demonstrates a proteolytic effect and can be used for disinfection. To get its bactericidal effect, NaOCl is highly dependent on the length of time it is retained in the canal and the use of copious volumes of solution. ^13^ Irrigating solution containing Chlorhexidine may also be used as an alternative because of its superior intracanal antimicrobial effect. ^9^ However, both NaOCl and Chlorhexidine can not perfectly eliminate *E. faecalis*, ^14^ especially in a place where irrigating solutions cannot easily flush.

### Obturation

The process of root canal obturation is a time-consuming and costly manipulation. In this process, an apical seal is crucial. ^15^ In a comparative study of 3 obturation techniques, the thermafil obturation technique showed better results in terms of voids and gaps found between gutta-percha and canal walls at the apical third of root canals compared to warm vertical condensation. The cold lateral obturation technique showed more voids and gaps than the other two. ^16^ However, none of the established techniques for root canal filling we have known today can guarantee a perfect seal. ^17^

In single-visit endodontic treatment, the whole process could be done in one visit. In multiple-visit endodontic treatment, patients will have more than one appointment in which inter-appointment intracanal medicament is used to improve disinfection before obturation. Calcium hydroxide has been the gold standard for intracanal medicament to fight against root canal pathogens. Combining calcium hydroxide with 2% Chlorhexidine gives better results in terms of percentage resolution of periapical radiolucency. ^18^ However, the penetration depth of both conventional and nanoparticle calcium hydroxide to dentinal tubules is low in the apical zone. ^19^

## Trepanation: bringing back oxygen circulation to a nonvital tooth

There is still a question of whether single-visit root canal treatment is adequate for eliminating root canal bacteria that can cause future reinfection. ^20^ However, multiple-visit root canal treatment is not preferable for patients, not only during the COVID-19 pandemic. Bacteria that are difficult to clean in the root canal, particularly in dentinal tubules, can live in an anaerobic environment. The failure of root canal treatment generally involves the growth of anaerobes and facultative anaerobes that become pathogenic and more efficient to infect in the absence of oxygen. ^21^^–^^24^

Some strategies to avoid reinfection after root canal treatment are preventing coronal microleakage that inadequate temporary or permanent fillings might cause, and eliminating bacteria that reside in dentinal tubules. Therefore, besides ensuring that no saliva, fluids, microorganisms, and debris can enter the coronal microleakage, we must also consider the antimicrobial agent we use and its delivery system. Disinfection must be able to reach the dentinal tubules where some bacteria can live and survive anaerobically. ^22^ Dental pain can occur in nonvital teeth, including after root canal treatment, and is often a symptom of an abscess. Most dental abscess treatments using antibiotics focus on attacking anaerobes and facultative anaerobes. ^25^ Endodontic infections are polymicrobial, with obligate anaerobic bacteria undeniably dominating the microorganism in primary infections. ^26^ NaOCl is mentioned by numerous researches to be effective against polymicrobial root canal biofilms. ^27^^,^^28^ Virgin coconut oil is also believed to have antiprotozoal, antiviral, and antibacterial properties, ^29^^,^^30^ but to use it as an irrigating solution still needs further studies. Understanding on how oxygen can make a difference in the root canal (please see Table 1) is also an attempt to harness its use in preventing endodontic infection that is dominated by obligate anaerobes.

**Table 1.** Oxygen and its relation to the root canal.

**Comparison of the size of oxygen molecule versus dentinal tubules diameter:** 0,299 nm, ^31^ versus not smaller than 0,4 m. ^32^ **Influence of oxygen on anaerobes:** Obligate anaerobes cannot tolerate and surmount the stress that oxygen creates. Oxygen is toxic for obligate anaerobes, and they cannot grow in the presence of oxygen. ^33^^,^^34^ **Influence of oxygen on facultative anaerobes:** Facultative anaerobes can tolerate oxygen and are well adapted to cellular hypoxia; they are also the most life-threatening pathogenic. 8 of the 12 priority pathogens in the WHO antibiotic-resistant pathogens list are facultative anaerobes. Under anaerobic conditions, the invasion efficiency of facultative anaerobe is increased. ^21^ Oxygen is needed to prevent the environment from becoming anaerobic.

Because the infection of the root canal of nonvital teeth tends to happen in an anaerobic condition, dental practitioners can consider preventing the root canal microenvironment from being oxygen-free. At some point, creating ventilation to avoid the absence of oxygen might look similar to trepanation. The size of the oxygen molecule is smaller than the diameter of dentinal tubules, making it able to fill the entire space available within the tooth. Oxygen can inhibit the growth of anaerobes and prevent facultative anaerobes from becoming more infectious. Using oxygen as an antimicrobial agent that fills the root canal is expected to shorten the working time of a dentist performing root canal treatments. It can replace conventional obturation after proper cleaning and shaping.

Oxygen can reach the anaerobic microenvironment in dentinal tubules at the apical third in which irrigating solutions cannot easily flush. This action will disturb the lives of anaerobes. However, facultative anaerobes are still possible to be found in the dentinal tubules and cannot be eliminated by oxygen. *Enterococcus faecalis* is the most discussed facultative anaerobe in endodontic infection and can colonize dentinal tubules to a depth of >1000 μm. ^35^ Not always pathogenic, *Enterococcus faecalis* can also be helpful and might be considered a potential probiotic, as shown in a study of *E. faecalis* in human milk. ^36^ If it is very challenging to eliminate *E. faecalis* perfectly, even by using any recommended irrigating solutions and special techniques, then perhaps the next strategy is to make them probiotic (mutualism), or at least nonpathogenic and nondestructive (commensalism). Oxygen might play its role in preventing the increase of their invasion efficiency and pathogenicity in the case where cleaning and shaping are not adequate enough to eliminate *E. faecalis* in the root canal system. Beside its nature that can be a commensal, *Enterococcus* spp. is also prominent for probiotic candidacy because of its potential antibacterial, antifungal, and antiviral activity. Its ability to produce bacteriocins is essential for these activities. ^37^

Trepanation was originally known as an act of perforating the skull. It might be the oldest surgical procedure, performed by many civilizations in the past, from Greece to China, and used in western medicine. ^38^ In modern medicine, surgeons still use this method through minimally invasive trepanation and drainage. It is also considered highly effective for treating purulent meningitis, ^39^ and its use in dentistry is no exception. Dental trepanation is a simple procedure of perforating the pulp chamber and keeping it open without any medicament inserted into the pulp. It is not easy to find academic literature that discusses this procedure, but it cannot be the reason to pretend as if it has never existed. Dental trepanation in a nonvital tooth is not only to let the pus from dental or periapical abscess out but also to prevent abscess formation. Oxygen can fill inside the tooth and eliminate the bacteria that can cause the abscess. A case report of a 12-year-old child in Indonesia showed that a dental abscess on a permanent maxillary lateral incisor improved after being given antibiotics and trepanation by making a hole that penetrates the pulp chamber. The patient did not come as instructed on the following third to seventh day. However, after 45 days, the tooth’s general conditions were good and there were no complaints. Neither there were any symptoms of dental abscess despite the fact that the use of antibiotics had been completed for a long time and no medicament was placed inside the pulp chamber. ^40^ Dental abscess might exist when a tooth is nonvital in which the blood cannot circulate and bring oxygen to the tooth anymore. In this case, dental trepanation can be seen as a method to bring back oxygen circulation to a nonvital tooth.

A study that measured the oxygen saturation level of a dental pulp found that the oxygen saturation level will be decreased when the pulp is in an unfavorable condition. Healthy teeth had the highest oxygen saturation level (94.6%), while reversible pulpitis, irreversible pulpitis, and pulpal necrosis were 85.4%, 81.6%, and 70.7%, respectively. ^41^ There is a question from us about the oxygen saturation level of pulpal necrosis; whether it was measured on necrotic teeth with or without pulp exposure (e.g., nonvital teeth due to trauma). However, we can still conclude that the more severe the pathological state of the pulp, the lower the oxygen saturation level is.

Another interesting thing to discuss is the comparison between secondary caries and inactive caries. Cariogenic bacteria in saliva that enter through microcracks with a width of at least 50 μm between fillings and dental tissue are considered to cause secondary caries if they grow in an environment that suits them. ^42^ On the other hand, caries can become inactive and do not develop for at least 4 to 5 years of follow-up in 85-90% of cases, making it not need any specific treatment, including restoration. ^43^ In inactive caries, saliva certainly has no significant obstacle to contacting the tooth surface because no filling or anything is covering, but the bacteria are still not easy to progress. Suppose both secondary caries and inactive caries are exposed to saliva. In that case, the difference is that in inactive caries, the surface is more open and easier to clean to avoid debris adhesion. Inactive caries that do not develop for years is one reason to expect oxygen circulation can prevent infection in the root canal that priorly has been cleaned from various obstruction and contamination.

## Methods: novel membranous dental restoration system

Dental trepanation has been known by some, if not many, dental practitioners in Indonesia. It is also believed that the method still exists until recently. If this conventional method of dental trepanation - done without being followed by any modern technique of root canal preparation - was once considered beneficial for nonvital teeth, we hypothesize that a thoroughly cleaned, disinfected, and completely dried root canal, sealed with a specific restoration that uses an oxygen-permeable membrane, will give a better result. The membrane will let oxygen in while fluid, debris, and microorganisms cannot enter the root canal. The oxygen that keeps circulating to the entire root canal system and dentinal tubules will act as an antimicrobial agent that is constantly renewed to prevent the growth of pathogens inside the tooth.

**Figure 1.**
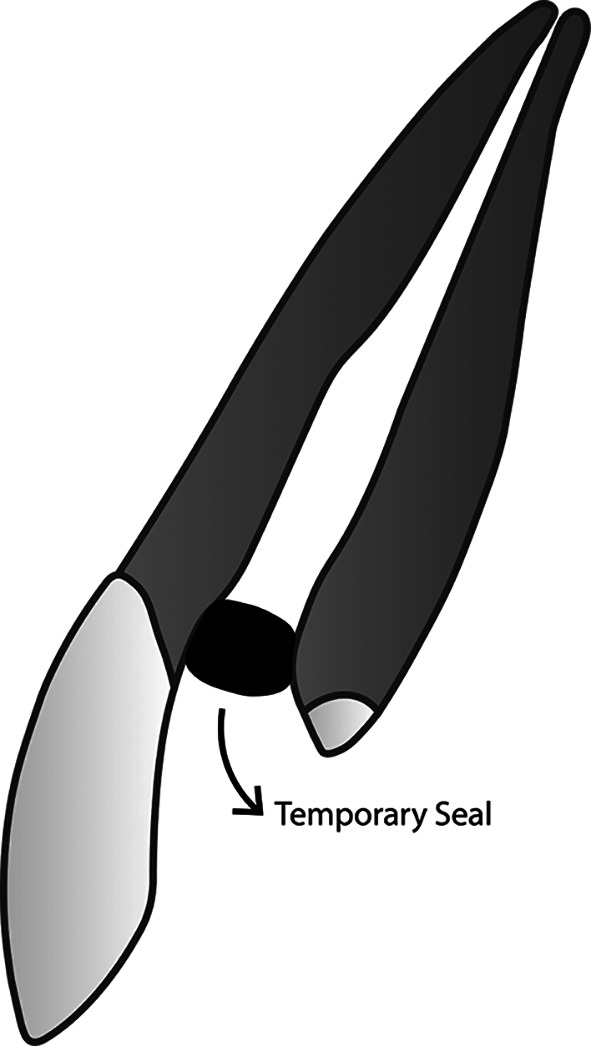
(This figure is an original figure produced by the authors for this article). The aseptic technique should always be applied during the whole restoration process. Access to the root canal should be covered tightly to prevent debris and fluid from entering the clean and dry root canal. A small piece of rubber, taken from a rubber dam sheet, for example, can be used to seal the access to the root canal. Once the access is closed, tooth preparation can be performed.

**Figure 2.**
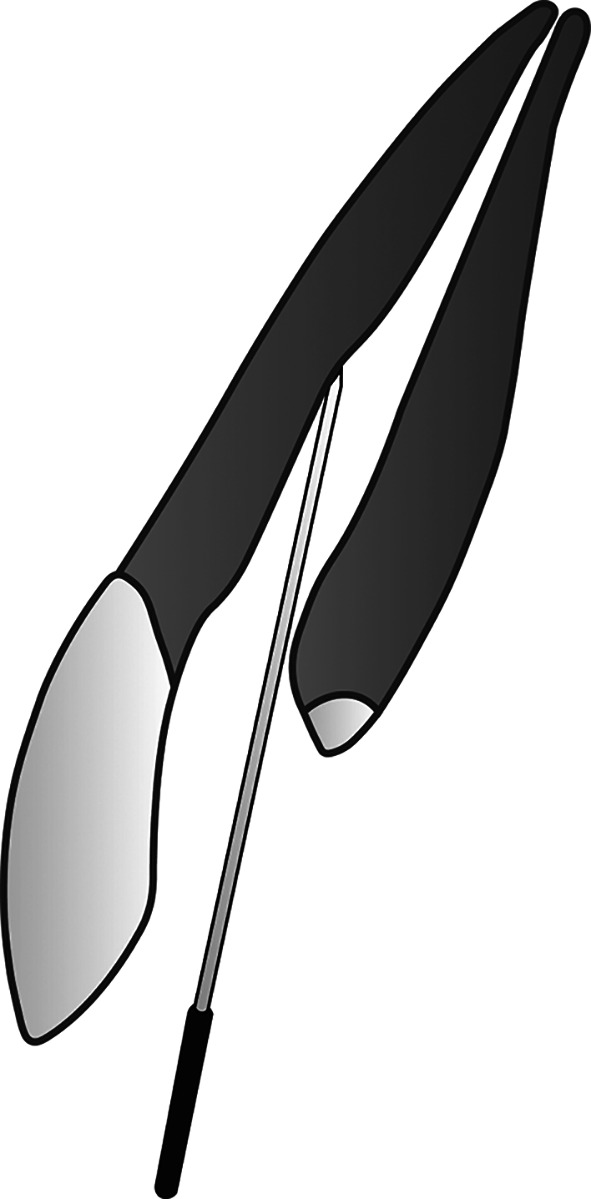
(This figure is an original figure produced by the authors for this article). Open the blockage made in the root canal after finishing any procedure needed for composite filling, then insert a sterile smooth surface miller needle (smooth broach) into the root canal. The depth does not have to be the same as the working length.

**Figure 3.**
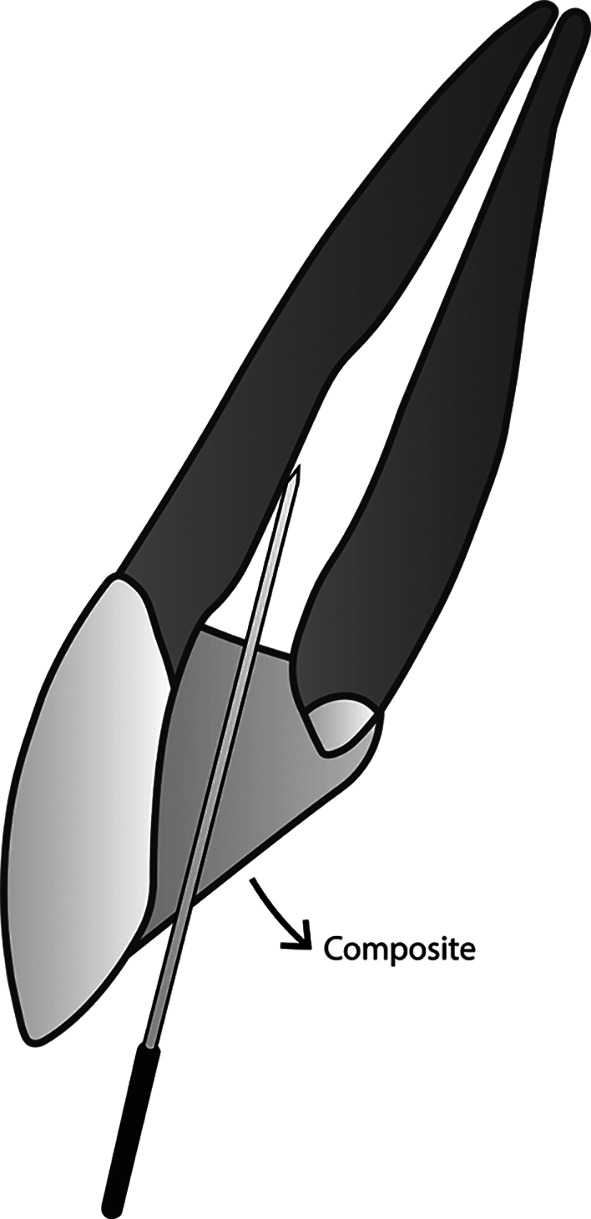
(This figure is an original figure produced by the authors for this article). A sufficient amount of composite is placed while keeping aware that the smooth broach must still be able to create a duct that connects the root canal system with the free air outside of the restoration.

**Figure 4.**
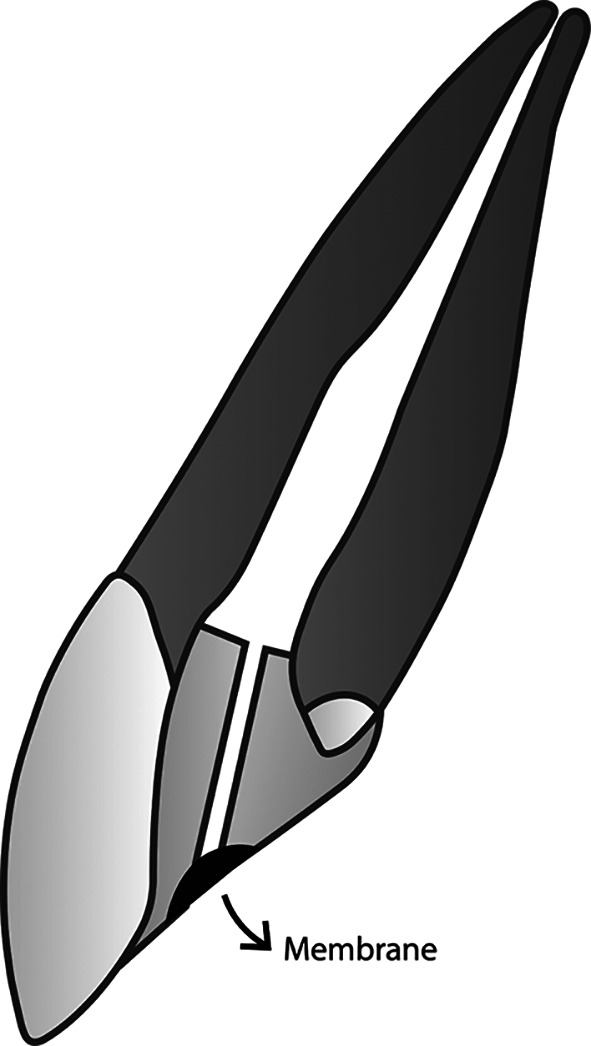
(This figure is an original figure produced by the authors for this article). The smooth broach is pulled out gently by twisting it, and the location to place the membrane in the restoration is right at the orifice of the duct.

The novel membranous restoration system described below (please see Figures 1 – 4) is expected to decrease the working time in endodontic treatment, as it can replace the time-consuming conventional obturation techniques. Any conventional sealer for obturation, including those that use nanoparticles, and any established technique, cannot give a perfect seal in the root canal system. Naturally, oxygen can flow automatically and fill any space it can infiltrate. Using oxygen as obturation biomaterials will hopefully be easier and more time-saving than all current obturation techniques that use solid substances. Before applying this restoration design, proper root canal preparation, including debridement and disinfection, should be done first.

### Tooth with multiple roots

In the case of a tooth with multiple roots, the duct shaped by the smooth broach must meet and be connected to all of the root canals.

### Smooth broach bending

A smooth broach can be bent to make it possible to adjust the direction of the duct in accordance with what we have designed. The orifice of the duct can be placed on the surface where fluid and debris will be difficult to accumulate and is easy to clean when brushing teeth. Any other bendable needle, such as a finger spreader, can also be used as long as the size is suitable, can be pulled out from the restoration, and can be sterilized before inserting it to the root canal.

### Noncomposite restorations

Using noncomposite materials is still possible as long as a small diameter duct is provided and connects the entire root canal with free air outside the restoration.

### The use of membrane

The oxygen-permeable membrane placed at the orifice of the duct is used to prevent any fluid, debris, and microorganisms from entering the root canal while letting oxygen go through. The membrane must also be of clinically safe material, durable to be placed in the mouth, and can be firmly attached to the selected restoration material. It should be noticed that the membrane must not be placed when the root canal is not clean and dry yet as the membrane cannot be passed by any fluid, including pus and irrigation solutions. In this case, it might be even better to use a membrane that can meet the following two criteria: it can let the gas flow from both sides of the membrane (two-way), and it can also let the fluid go through only from one side of the membrane (one-way).

Membranes made of silanized alumina are both highly permeable to oxygen and hydrophobic. Without silanization, these membranes are hydrophilic. If it is possible to create a hydrophobic surface on one side and a hydrophilic surface on the other, we speculate that the same concept may apply to dental restoration. The oxygen-permeable membrane has never been utilized in the field of dentistry. However, silanized alumina membranes are claimed to be attractive for use in several technological disciplines, including oxygenation of blood during open-heart surgery. ^44^

### Clinical case example: long-term trepanation

As previously mentioned, trepanation can be used in surgery to address purulent infections by providing a drainage pathway. ^39^ Trepanation is often temporary, for instance, in cases of dental abscesses to alleviate swelling, although patients may feel comfortable for a longer period than the time frame given by the dentist for a return visit to the clinic. ^40^ While trepanation is formally utilized in both the pediatric dentistry department and the endodontic department at a dental hospital in Bandung, Indonesia, ^45^ there is still a lack of publications specifically discussing the use of this procedure, especially those presented in English.

A case report, possibly the only publication to date describing the clinical application of trepanation, can be used as an example of trepanation in a dental case. The article discusses an avulsed tooth case involving the upper left central incisor of a 13-year-old boy. ^46^ As described in the report, avulsion cases present unique challenges compared to cases of dental necrosis that do not involve avulsion. Trepanation was performed on the patient due to concerns about the potential development of an abscess, as the avulsed tooth had been at risk of contamination after being on the ground for an extended period in a non-sterile condition. The report also highlights a phenomenon of long-term trepanation, where the patient remained comfortable for over two years without any complaints and could engage in regular activities, even forgetting to schedule follow-up appointments with the dentist. The radiographic findings after two years revealed no signs of an abscess. This indicates that long-term trepanation can provide comfort to the patient, serving not only as a drainage effort for an existing abscess but also to prevent the formation of abscesses predominantly caused by anaerobes.

From the case report, we conclude that the concept of trepanation holds considerable promise for further exploration, not only for short-term treatments such as abscess drainage. The development of an alternative method may be pursued if it demonstrates superiority over existing techniques. Should the alternative method present potential benefits while carrying risks that are relatively comparable to those of established methods, researchers may still continue to investigate it, particularly if it is found to be faster, easier, and more cost-effective to implement. Although this study is not intended to compare various methods that can be used in endodontic treatment, the use of a gas that flows easily as a filling material for root canals logically appears to be quicker, simpler, and less expensive than the use of solid filling materials.

The challenges we observe in the case report primarily revolve around concerns regarding food particles that may enter and obstruct the trepanation channel, necessitating that patients maintain strict hygiene in the area. Utilizing a covering material with oxygen-permeable properties is a potential solution to address the challenges that may arise with traditionally performed trepanation.

## Conclusion

Because of the complexity of the root canal system and the tiny size of dentinal tubules, it is very complicated, if not impossible, to disinfect and obturate them entirely by using any recommended and established techniques. Endodontic treatment seems to require much time and multiple visits to deal with this challenge, which is unfavorable, especially during the pandemic. A new perspective is needed to reduce the working time as well as to answer some unsolved problems in endodontic treatment. The expected results of the development of this novel membranous dental restoration system in the future is not only to deal with microorganisms in a nonvital tooth, including *Enterococcus faecalis*, but also to find another way to fill the root canal system completely. Disinfecting the hard-to-kill *E. faecalis* and performing complex obturation techniques are somehow tiring and time-consuming. Oxygen is hoped to be the agent to prevent *E. faecalis* from being pathogenic and fill the root canal system with ease.

In this article, a dental restoration system that uses a membrane to provide oxygen to the root canal system is a somewhat novel idea we would like to introduce to the field of dentistry. By publishing the concept and making it available to the research community, any discussion might be useful to accelerate the exploration of the use of oxygen-permeable membrane for dental restoration. The function of the oxygen-permeable membrane in the restoration design is more or less similar to the medical mask used during the COVID-19 pandemic, as it enables the oxygen to circulate while preventing any fluid, debris, and microorganisms from passing. Further research is needed to find the most suitable membrane for dental restoration that allows the oxygen circulation at the utmost, and how effective this method can prevent infection. We are also open to collaborate with other researchers to implement the concept described in this article.

## Data Availability

No data are associated with this article.
