# Oxygen as obturation biomaterial in endodontic treatment: development of novel membranous dental restoration system

**DOI:** 10.12688/f1000research.132479.3

**Published:** 2024-12-09

**Authors:** Didi Wahyudi, Citra Kusumasari

**Affiliations:** 1 Center of Excellence Biomedical and Healthcare Technology, Telkom University, Bandung, Indonesia; 2 Dental Cooperation Indonesia, Bandung, 40134, Indonesia; 3 Department of Conservative Dentistry, Faculty of Dentistry, Universitas Indonesia, Depok, West Java, 16424, Indonesia

**Keywords:** dental restoration system, Enterococcus faecalis, obturation biomaterials, oxygen permeable membrane, endodontics treatments; trepanation

## Abstract

Complexities in obturation and difficulties in disinfection represent significant issues that render endodontic treatment notably time-consuming. A new perspective is essential to reduce both working time and address these two challenges. To date, none of the established techniques for root canal obturation can assure a perfect seal. Solid materials are not easily manipulated to hermetically fill the intricate branches of the root canal system. Concurrently, anaerobic and facultative anaerobic bacteria, particularly *Enterococcus faecalis*, are predominant in discussions surrounding endodontic infections. Numerous studies have demonstrated that achieving complete disinfection of *Enterococcus faecalis* is exceedingly difficult, even with the use of irrigating solutions that possess strong antibacterial properties. Under anaerobic conditions, the invasion efficiency of facultative anaerobes is heightened. If irrigation and disinfection fail to entirely eliminate anaerobes and facultative anaerobes, a novel strategy is required to address the bacteria that persist within the root canal. Oxygen can easily permeate the root canal system, eradicate anaerobes, and inhibit facultative anaerobes from becoming pathogenic. Therefore, employing oxygen as a biomaterial for obturation following appropriate cleaning and shaping procedures is anticipated to address the two primary endodontic issues. This article aims to explore a new potential concept for a dental restoration system that utilizes an oxygen-permeable membrane to reduce the time required for endodontic treatment. The membrane is positioned at the orifice of a duct designed to connect the entire root canal system with ambient air outside the restoration. The function of the membrane is somewhat analogous to the masks used during the COVID-19 pandemic, as it allows for the circulation of oxygen while preventing the passage of fluids, debris, and microorganisms. We hypothesize that the oxygen circulating within the root canal system will also function as a continuously renewing antimicrobial agent.

## Introduction

Some variants of concern of severe acute respiratory syndrome coronavirus 2 (SARS-CoV-2) or coronavirus disease 2019 (COVID-19) have been recorded. ^1^ Dentists are particularly susceptible to COVID-19 infection due to their proximity to a patient’s mouth during treatment. Saliva can harbour various viruses, including coronaviruses, ^2^ and is an element that dentists cannot entirely avoid when performing any procedure in the oral cavity. Therefore, both dentists and patients must exercise caution when providing or receiving dental care.

Various measures have been implemented to address the spread of COVID-19 in dental practice, ranging from the closure of dental clinics to the restriction of certain types of treatments that can only be performed under stringent protocols. ^3^ The rationale behind all these decisions is to minimize or reduce interactions with patients who may potentially transmit the coronavirus. Conversely, patients are making efforts to avoid visiting locations they perceive as potentially contaminated with the virus, such as hospitals and dental clinics. Thus far, the COVID-19 pandemic has significantly influenced the manner in which dentists operate.

Endodontic treatment typically necessitates multiple visits, which can pose a disadvantage for patients. Nevertheless, root canal therapy may still be essential for individuals experiencing severe dental pain, as most emergency cases in dentistry require endodontic intervention. ^4^ In a study conducted in India during the COVID-19 pandemic, three primary reasons for emergency visits to dental clinics were identified: pulpal problems at 46.0%, abscesses at 16.6%, and periapical lesions at 15%. All of these issues were indeed related to endodontic concerns. ^5^

The pandemic has motivated dentists to enhance their efficiency, particularly in resolving patient cases quickly. This drive can persist beyond the pandemic, as streamlining treatment remains advantageous for patients. This article aims to explore a new potential concept, previously undiscussed in any scientific journals, to reduce the time required for endodontic treatment.

## Current endodontic treatment

There are ten principles to consider in endodontic treatment. The first and second principles are aseptic technique and the restriction of instruments to the root canal. Third, root canal preparation should be conducted using appropriate instruments. Fourth, the root canal should be expanded, if feasible, to facilitate cleaning. Fifth, the root canal must be thoroughly irrigated with antiseptic solutions. Sixth, any irrigating solutions used must be safe for periapical tissues. Seventh, if a sinus tract is present, it should be addressed following root canal therapy and does not necessitate surgery. An incision in the soft tissue may be performed for an acute periapical abscess to allow for drainage. Eighth, the root canal should be hermetically obturated to ensure a proper seal. Ninth, prior to obturation, a negative culture should be obtained. Tenth, all materials used in obturation must be biocompatible. These principles are based on the conclusions drawn at the International Conference on Endodontics in 1958. ^6^^–^^8^

Successful root canal therapy depends on three key factors: cleaning and shaping, disinfection, and obturation. ^9^ Periradicular pathosis is primarily caused by the growth of pathogens in the root canal system, and unsuccessful treatment is not directly linked to errors in the procedure itself. ^10^

### Cleaning and shaping

Cleaning and shaping are separate and distinct concepts, yet they are performed concurrently. Some of the most significant factors influencing the outcome of the cleaning process include the anatomy and morphology of the tooth, as well as the instruments and irrigants available during the procedure. The primary objective of shaping is to maintain or develop a continuously tapering funnel from the orifice to the apex. Irritants should be minimized as much as possible, if not entirely eliminated. An adequately prepared root canal should feel smooth in all dimensions when the tip of a small file is pushed against the canal wall. Following cleaning and shaping, sufficient space should be available for the placement of obturation materials. Irrigation with 17% ethylenediaminetetraacetic (EDTA) for one minute is recommended to remove the smear layer that accumulates on the radicular canal wall. ^11^ The success of root canal therapy is primarily achieved through proper cleaning and shaping. Endodontically treated teeth fail not due to inadequate obturation, but rather as a result of insufficient cleaning and shaping. ^9^^,^^12^

### Disinfection

Most clinicians opt for sodium hypochlorite (NaOCl) due to its proteolytic properties and its efficacy in disinfection. The bactericidal effect of NaOCl is significantly influenced by the duration it remains in the canal and the application of large volumes of the solution. ^13^ Irrigating solutions containing Chlorhexidine may also be utilized as an alternative due to their superior antimicrobial effect within the canal. ^9^ However, both NaOCl and Chlorhexidine cannot perfectly eliminate *E. faecalis*, ^14^ especially in areas where irrigating solutions cannot be easily flushed.

### Obturation

The process of root canal obturation is a time-consuming and costly manipulation. In this process, achieving an apical seal is crucial. ^15^ In a comparative study of three obturation techniques, the thermafil obturation technique demonstrated superior results regarding the presence of voids and gaps between gutta-percha and canal walls in the apical third of root canals when compared to warm vertical condensation. The cold lateral obturation technique exhibited more voids and gaps than the other two. ^16^ However, none of the established techniques for root canal filling known today can guarantee a perfect seal. ^17^

In single-visit endodontic treatment, the entire procedure can be completed in one appointment. In contrast, multiple-visit endodontic treatment requires patients to attend more than one appointment, during which inter-appointment intracanal medicament is utilized to enhance disinfection prior to obturation. Calcium hydroxide has long been regarded as the gold standard for intracanal medicament in combating root canal pathogens. The combination of calcium hydroxide with 2% Chlorhexidine yields improved outcomes regarding the percentage resolution of periapical radiolucency. ^18^ However, the penetration depth of both conventional and nanoparticle calcium hydroxide into dentinal tubules is low in the apical zone. ^19^

The use of solid materials for filling root canals, inherent in all obturation techniques, presents a common limitation: the challenge of achieving a hermetic seal in narrow and convoluted spaces within the root canal. This limitation provides the rationale for considering non-solid materials for filling root canals. This article explores a strategy to tackle two common challenges in endodontic treatment: complexities in obturation and difficulties in disinfection. Using a flowing gas to fill narrow root canal spaces aims to enhance obturation, while selecting a safe gas that inhibits pathogen growth is expected to improve disinfection. Oxygen, with its natural ability to flow into narrow and convoluted spaces, can be regarded as a potential obturation biomaterial in endodontic treatment.

## Trepanation: bringing back oxygen circulation to a nonvital tooth

There remains a question regarding the adequacy of single-visit root canal treatment in eliminating root canal bacteria that may lead to future reinfection. ^20^ However, multiple-visit root canal treatment is generally not preferred by patients. Bacteria that are challenging to eliminate from the root canal, particularly within dentinal tubules, can survive in an anaerobic environment. The failure of root canal treatment typically involves the proliferation of anaerobes and facultative anaerobes that become pathogenic and more adept at causing infection in the absence of oxygen. ^21^^–^^24^

Some strategies to avoid reinfection after root canal treatment include preventing coronal microleakage that may result from inadequate temporary or permanent fillings, as well as eliminating bacteria residing in dentinal tubules. Therefore, in addition to ensuring that no saliva, fluids, microorganisms, or debris can enter through coronal microleakage, dentists must also consider the antimicrobial agent utilized and its delivery system. Disinfection must effectively reach the dentinal tubules where certain bacteria can live and survive anaerobically. ^22^ Dental pain can occur in nonvital teeth, including following root canal treatment, and is often indicative of an abscess. Most treatments for dental abscesses utilizing antibiotics focus on targeting anaerobic bacteria and facultative anaerobes. ^25^ Endodontic infections are polymicrobial, with obligate anaerobic bacteria undeniably dominating the microorganism in primary infections. ^26^ NaOCl is mentioned by numerous researches to be effective against polymicrobial root canal biofilms. ^27^^,^^28^ Virgin coconut oil is also believed to have antiprotozoal, antiviral, and antibacterial properties, ^29^^,^^30^ but to use it as an irrigating solution still needs further studies. Understanding on how oxygen can make a difference in the root canal (please see Table 1) is also an attempt to harness its use in preventing endodontic infection that is dominated by obligate anaerobes.

**Table 1.** Oxygen and its relation to the root canal.

**Comparison of the size of oxygen molecule versus dentinal tubules diameter:** 0,299 nm, ^31^ versus not smaller than 0,4 m. ^32^ **Influence of oxygen on anaerobes:** Obligate anaerobes are unable to tolerate and overcome the stress induced by oxygen. Oxygen is toxic to obligate anaerobes, and they cannot proliferate in its presence. ^33^^,^^34^ **Influence of oxygen on facultative anaerobes:** Facultative anaerobes can tolerate oxygen and are well adapted to cellular hypoxia; they are also among the most life-threatening pathogenic. Eight of the twelve priority pathogens listed by the WHO as antibiotic-resistant are facultative anaerobes. Under anaerobic conditions, the invasion efficiency of facultative anaerobes is increased. ^21^ Oxygen is essential to prevent the environment from becoming anaerobic.

Because the infection of the root canal in nonvital teeth tends to occur in anaerobic conditions, dentists may consider preventing the root canal microenvironment from becoming oxygen-free. At times, creating ventilation to avoid the absence of oxygen may resemble trepanation. The size of the oxygen molecule is smaller than the diameter of dentinal tubules, allowing it to fill the entire space within the tooth. Oxygen can inhibit the growth of anaerobes and prevent facultative anaerobes from becoming more infectious. Utilizing oxygen as an antimicrobial agent to fill the root canal is expected to reduce the working time for dentists performing root canal treatments. It can serve as a replacement for conventional obturation following proper cleaning and shaping.

Oxygen can penetrate the anaerobic microenvironment within the dentinal tubules at the apical third, where irrigating solutions are unable to effectively flush. This presence of oxygen disrupts the existence of anaerobic organisms. However, facultative anaerobes may still be present in the dentinal tubules and cannot be eradicated by oxygen. *Enterococcus faecalis* is the most frequently discussed facultative anaerobe in endodontic infections and has the ability to colonize dentinal tubules to a depth exceeding 1000 μm. ^35^ Not always pathogenic, *Enterococcus faecalis* can also be beneficial and may be regarded as a potential probiotic, as demonstrated in a study of *E. faecalis* in human milk. ^36^ Given the challenge of completely eradicating *E. faecalis*, even with recommended irrigants and techniques, an alternative strategy can be to enhance its probiotic potential (mutualism) or ensure it remains nonpathogenic and nondestructive (commensalism). Oxygen might help limit its invasion efficiency and pathogenicity when cleaning and shaping fail to eliminate *E. faecalis* from the root canal. Apart from its commensal attributes, *Enterococcus spp.* is also a promising probiotic candidate due to its antibacterial, antifungal, and antiviral properties, largely due to its ability to produce bacteriocins. ^37^

Trepanation was originally defined as the act of perforating the skull. It may be the oldest surgical procedure, practiced by numerous civilizations throughout history, from Greece to China, and has been utilized in Western medicine. ^38^ In modern medicine, surgeons continue to employ this method through minimally invasive trepanation and drainage. It is also regarded as highly effective for treating purulent meningitis, ^39^ and its application in dentistry is no exception. Dental trepanation is a straightforward procedure involving the perforation of the pulp chamber while keeping it open without the introduction of any medicament into the pulp. Academic literature discussing this procedure is scarce, but this should not lead to the misconception that it has never existed. The purpose of dental trepanation in a nonvital tooth extends beyond merely allowing the drainage of pus from dental or periapical abscesses; it also serves to prevent the formation of abscesses. The introduction of oxygen into the tooth can help eradicate the bacteria responsible for abscess development. A case report involving a 12-year-old child in Indonesia indicated that a dental abscess on a permanent maxillary lateral incisor showed improvement following the administration of antibiotics and trepanation, which involved creating a hole that penetrated the pulp chamber. The patient did not return as instructed on the third to seventh day. However, after 45 days, the overall condition of the tooth was satisfactory, and there were no complaints. Furthermore, there were no signs of dental abscess, despite the completion of antibiotic treatment a considerable time prior and the absence of any medicament placed within the pulp chamber. ^40^ A dental abscess can occur when a tooth becomes nonvital, lacking blood circulation for oxygen supply. In such instances, dental trepanation can be seen as a method to bring back oxygen circulation to a nonvital tooth.

A study that measured the oxygen saturation level of dental pulp found that the oxygen saturation level decreases when the pulp is in an unfavourable condition. Healthy teeth exhibited the highest oxygen saturation level at 94.6%, while reversible pulpitis, irreversible pulpitis, and pulpal necrosis recorded levels of 85.4%, 81.6%, and 70.7%, respectively. ^41^ There is some uncertainty about the measurement of pulpal necrosis concerning whether it involved necrotic teeth with or without pulp exposure (e.g., nonvital teeth due to trauma). Nonetheless, it can be concluded that more severe pulp pathology correlates with lower oxygen saturation levels.

Another interesting topic of discussion is the comparison between secondary caries and inactive caries. Secondary caries are caused by cariogenic bacteria in saliva that enter through microcracks (at least 50 μm wide) between fillings and dental tissue, proliferating in a conducive environment. ^42^ On contrast, inactive caries can remain unchanged for 4 to 5 years in 85-90% of cases and typically do not require treatment, including restoration. ^43^ Inactive caries allow saliva to reach the tooth surface without obstruction, as there is no filling or covering, though bacteria do not easily progress. When saliva interacts with both types, inactive caries is more accessible and easier to clean, helping prevent debris adhesion, which can ultimately also benefit oxygen circulation. The stability of inactive caries suggests that oxygen circulation may aid in preventing infection in a previously cleansed root canal, free of obstructions and contaminants.

## Methods: novel membranous dental restoration system

Dental trepanation has been recognized by some, if not many, dental practitioners in Indonesia. It is also believed that this method has persisted until recent times. If this conventional technique of dental trepanation—performed without the subsequent application of any modern root canal preparation techniques—was once deemed beneficial for nonvital teeth, we hypothesize that a thoroughly cleaned, disinfected, and completely dried root canal, sealed with a specific restoration utilizing an oxygen-permeable membrane, will yield superior results. The membrane will allow oxygen to enter while preventing fluids, debris, and microorganisms from accessing the root canal. The oxygen that continuously circulates throughout the entire root canal system and dentinal tubules will function as an antimicrobial agent that is perpetually renewed to inhibit the growth of pathogens within the tooth.

**Figure 1.**
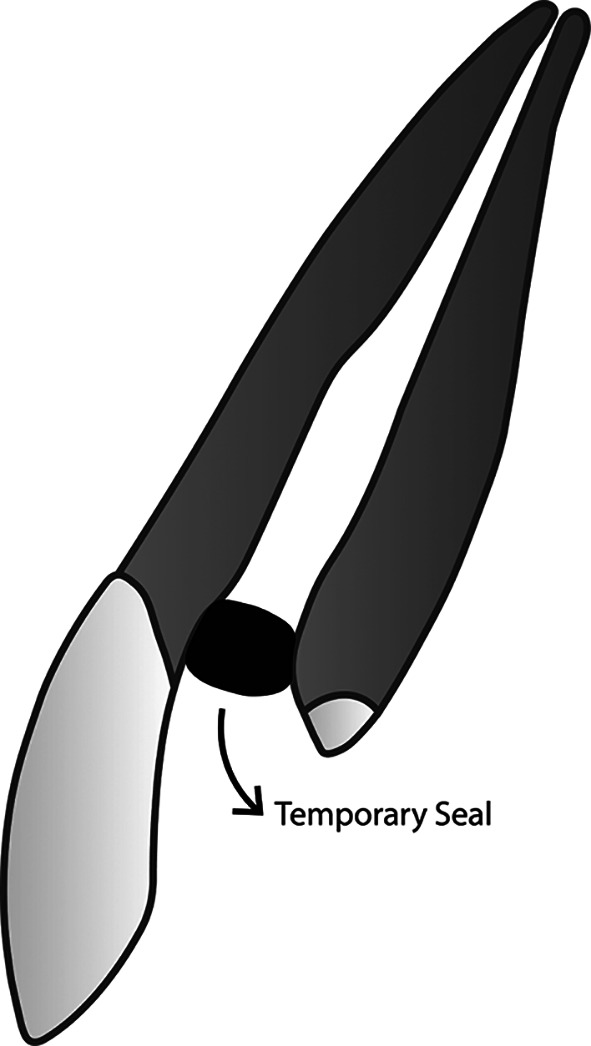
(This figure is an original figure produced by the authors for this article). The aseptic technique should always be applied during the whole restoration process. Access to the root canal should be securely sealed to prevent debris and fluids from entering the clean and dry root canal. A small piece of rubber, such as that taken from a rubber dam sheet, can be utilized to close the access to the root canal. Once the access is sealed, tooth preparation may proceed.

**Figure 2.**
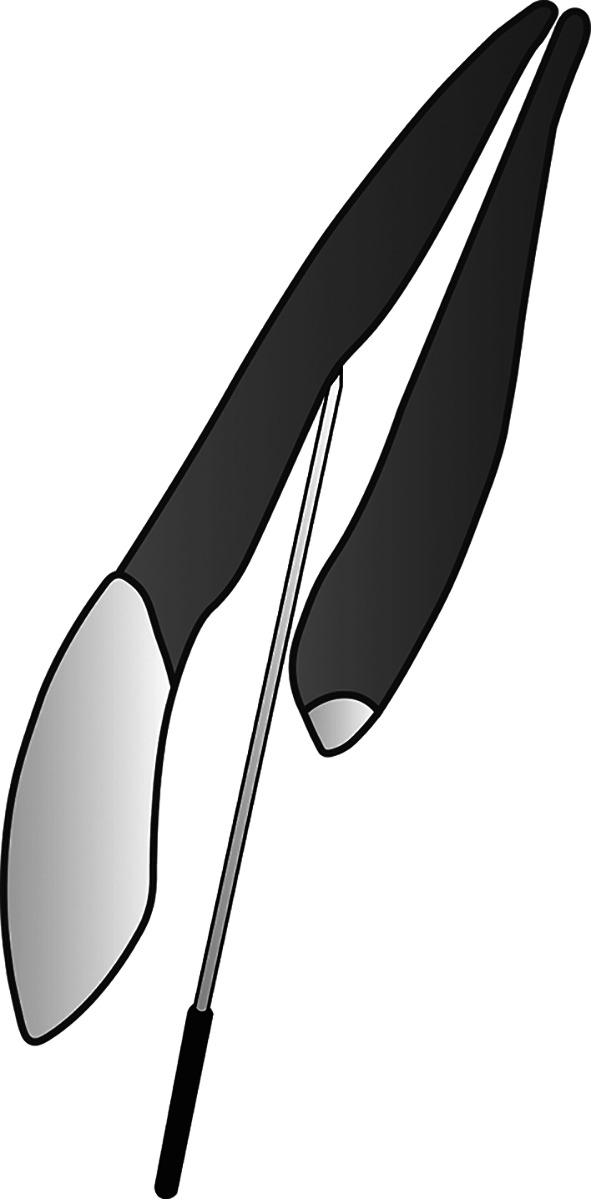
(This figure is an original figure produced by the authors for this article). Open the blockage created in the root canal after completing any necessary procedures for composite filling, then insert a sterile smooth surface miller needle (smooth broach) into the root canal. The depth does not need to match the working length.

**Figure 3.**
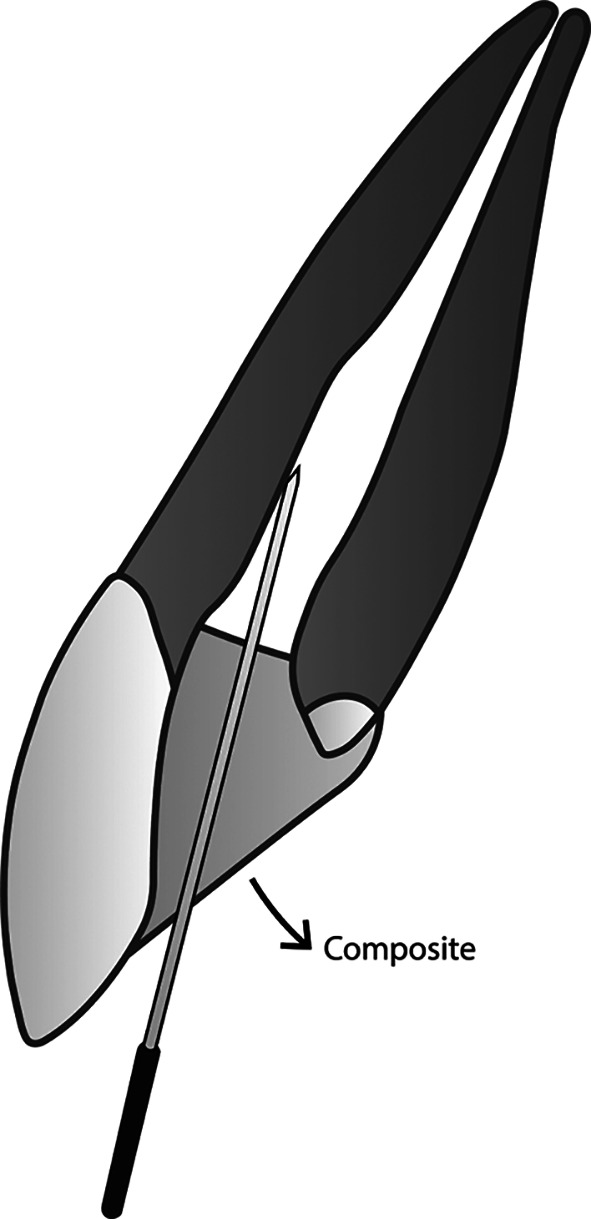
(This figure is an original figure produced by the authors for this article). A sufficient amount of composite material is applied, while ensuring that the smooth broach can still create a duct that connects the root canal system to the external air beyond the restoration.

**Figure 4.**
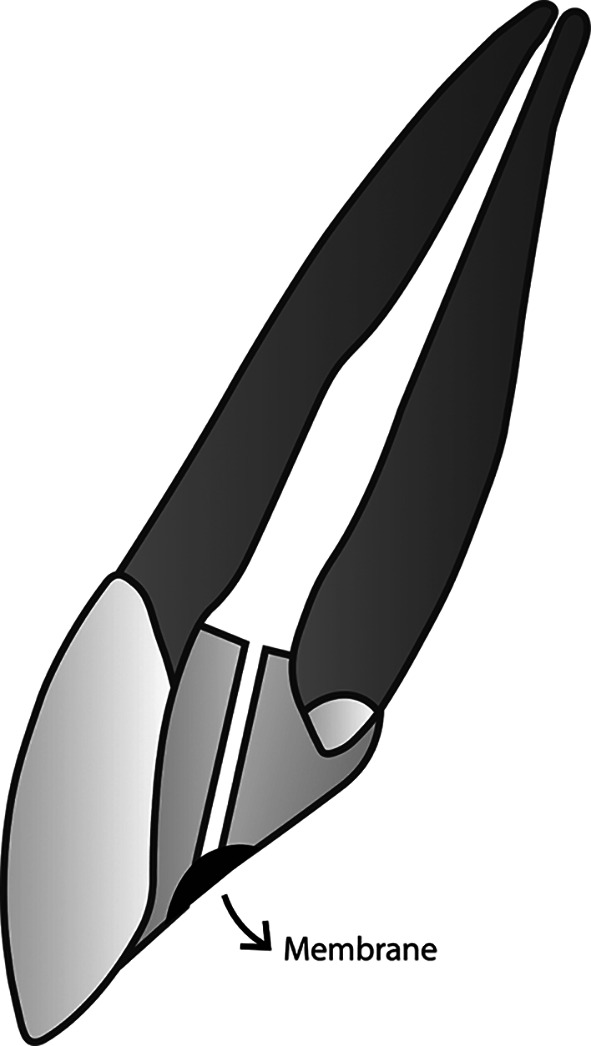
(This figure is an original figure produced by the authors for this article). The smooth broach is gently extracted by twisting it, and the appropriate location for placing the membrane in the restoration is directly at the orifice of the duct.

The novel membranous restoration system described below (please see Figures 1 – 4) is expected to decrease the working time in endodontic treatment, as it can replace the time-consuming conventional obturation techniques. Any conventional sealer for obturation, including those that incorporate nanoparticles, and any established technique, cannot achieve a perfect seal within the root canal system. Naturally, oxygen can flow freely and occupy any space it can infiltrate. Utilizing oxygen as an obturation biomaterial is anticipated to be easier and more time-efficient than all current obturation techniques that employ solid substances. Prior to implementing this restoration design, appropriate root canal preparation, including debridement and disinfection, must be conducted first.

### Tooth with multiple roots

In the case of a tooth with multiple roots, the duct shaped by the smooth broach must connect to all of the root canals.

### Smooth broach bending

A smooth broach can be bent to facilitate the adjustment of the duct’s direction. The orifice of the duct can be positioned on a surface where fluid and debris are less likely to accumulate, making it easier to clean during dental hygiene practices. Any other flexible instrument, such as a finger spreader, may also be utilized, provided that it is appropriately sized, can be removed from the restoration, and can be sterilized prior to insertion into the root canal.

### Noncomposite restorations

Using non-composite materials remains feasible, provided that a small diameter duct is established to connect the entire root canal with unobstructed air outside the restoration.

### The use of membrane

The oxygen-permeable membrane positioned at the orifice of the duct serves to prevent the ingress of any fluids, debris, and microorganisms into the root canal while allowing oxygen to pass through. The membrane must also be composed of clinically safe materials, durable enough for oral placement, and capable of being securely affixed to the chosen restorative material. It is important to note that the membrane should not be applied when the root canal is not yet clean and dry, as it cannot permit the passage of any fluids, including pus and irrigation solutions. In such instances, it may be preferable to utilize a membrane that fulfills the following two criteria: it should allow gas flow from both sides of the membrane (two-way), while also permitting fluid passage from only one side of the membrane (one-way).

Membranes composed of silanized alumina exhibit high permeability to oxygen and are hydrophobic in nature. In the absence of silanization, these membranes display hydrophilic properties. If it is feasible to engineer a hydrophobic surface on one side and a hydrophilic surface on the opposite side, we hypothesize that a similar principle could be applicable in the realm of dental restoration. The utilization of oxygen-permeable membranes has not yet been explored within the field of dentistry. Nevertheless, silanized alumina membranes are purported to be advantageous for application in various technological sectors, including the oxygenation of blood during open-heart surgery. ^44^

### Clinical case example: long-term trepanation

As previously mentioned, trepanation can be used in surgery to address purulent infections by providing a drainage pathway. ^39^ Trepanation is often temporary, for instance, in cases of dental abscesses to alleviate swelling, although patients may feel comfortable for a longer period than the time frame given by the dentist for a return visit to the clinic. ^40^ While trepanation is formally utilized in both the paediatric dentistry department and the endodontic department at a dental hospital in Bandung, Indonesia, ^45^ there is still a lack of publications specifically discussing the use of this procedure, especially those presented in English.

A case report, possibly the only publication to date describing the clinical application of trepanation, can be used as an example of trepanation in a dental case. The article discusses an avulsed tooth case involving the upper left central incisor of a 13-year-old boy. ^46^ As described in the report, avulsion cases present unique challenges compared to cases of dental necrosis that do not involve avulsion. Trepanation was performed on the patient due to concerns about the potential development of an abscess, as the avulsed tooth had been at risk of contamination after being on the ground for an extended period in a non-sterile condition. The report also highlights a phenomenon of long-term trepanation, where the patient remained comfortable for over two years without any complaints and could engage in regular activities, even forgetting to schedule follow-up appointments with the dentist. The radiographic findings after two years revealed no signs of an abscess. This indicates that long-term trepanation can provide comfort to the patient, serving not only as a drainage effort for an existing abscess but also to prevent the formation of abscesses predominantly caused by anaerobes.

From the case report, we conclude that the concept of trepanation holds considerable promise for further exploration, not only for short-term treatments such as abscess drainage. The development of an alternative method may be pursued if it demonstrates superiority over existing techniques. Should the alternative method present potential benefits while carrying risks that are relatively comparable to those of established methods, researchers may still continue to investigate it, particularly if it is found to be faster, easier, and more cost-effective to implement. Although this study is not intended to compare various methods that can be used in endodontic treatment, the use of a gas that flows easily as a filling material for root canals logically appears to be quicker, simpler, and less expensive than the use of solid filling materials.

The challenges we observe in the case report primarily revolve around concerns regarding food particles that may enter and obstruct the trepanation channel, necessitating that patients maintain strict hygiene in the area. Utilizing a covering material with oxygen-permeable properties is a potential solution to address the challenges that may arise with traditionally performed trepanation.

## Conclusion

Due to the intricate nature of the root canal system and the minuscule size of dentinal tubules, it is exceedingly challenging, if not impossible, to entirely disinfect and obturate them using any recommended and established techniques. Endodontic treatment appears to necessitate considerable time and multiple visits to address this issue. A new approach is required to reduce the working time and to resolve some unresolved challenges in endodontic treatment. The anticipated outcomes of developing this innovative membranous dental restoration system in the future aim not only to address microorganisms in a nonvital tooth, including *Enterococcus faecalis*, but also to discover an alternative method for completely filling the root canal system. Disinfecting the resilient *E. faecalis* and executing complex obturation techniques can be exhausting and time-intensive. It is hoped that oxygen will serve as the agent to inhibit *E. faecalis* from becoming pathogenic and facilitate the filling of the root canal system with ease.

In this article, we would like to introduce a somewhat novel idea to the field of dentistry: a dental restoration system that utilizes a membrane to provide oxygen to the root canal system. By publishing this concept and making it accessible to the research community, we hope that any ensuing discussions may facilitate the exploration of oxygen-permeable membranes for dental restoration. The role of the oxygen-permeable membrane in the restoration design is analogous to that of a medical mask used during the COVID-19 pandemic, as it allows for the circulation of oxygen while preventing the passage of fluids, debris, and microorganisms. Further research is necessary to identify the most suitable membrane for dental restoration that maximizes oxygen circulation and to evaluate the effectiveness of this method in preventing infection. We are also open to collaborating with other researchers to implement the concept described in this article.

## Data Availability

No data are associated with this article.
