# Supplementary material for: Oxygen as obturation biomaterial in endodontic treatment: development of novel membranous dental restoration system
Source: F1000Res. 2024 Dec 9;12:380. Originally published 2023 Apr 11. [Version 3] doi: 10.12688/f1000research.132479.3 (PMC11584453; doi:10.12688/f1000research.132479.3)
Supplement: Peer review history

# Reviewer report: Reviewer response for version 3

**DOI:** 10.5256/f1000research.175456.r347041

**Published:** 2024-12-19

**Authors:** Mohammed Zahedul Islam Nizami

**Affiliations:** 1 The Forsyth Institute, Harvard University, Cambridge, MA, USA

I do not have additional comment.

Is the rationale for developing the new method (or application) clearly explained?

Partly

Is the description of the method technically sound?

Partly

Are the conclusions about the method and its performance adequately supported by the findings presented in the article?

Partly

If any results are presented, are all the source data underlying the results available to ensure full reproducibility?

No

Are sufficient details provided to allow replication of the method development and its use by others?

Partly

Reviewer Expertise:

Cariology, preventive and restorative dentistry, dental biomaterials

I confirm that I have read this submission and believe that I have an appropriate level of expertise to confirm that it is of an acceptable scientific standard.

# Reviewer report: Reviewer response for version 2

**DOI:** 10.5256/f1000research.174308.r338341

**Published:** 2024-11-22

**Authors:** Tanay V Chaubal

**Affiliations:** 1 Periodontics, The University of Oklahoma Health Sciences Center College of Dentistry, OKLAHOMA CITY, Oklahoma, USA

I have no further comments.

Is the rationale for developing the new method (or application) clearly explained?

Yes

Is the description of the method technically sound?

Yes

Are the conclusions about the method and its performance adequately supported by the findings presented in the article?

Partly

If any results are presented, are all the source data underlying the results available to ensure full reproducibility?

Partly

Are sufficient details provided to allow replication of the method development and its use by others?

Yes

Reviewer Expertise:

Periodontics, Oral Implantology, Systematic reviews and meta-analysis, Bone regeneration, Nanoparticles.

I confirm that I have read this submission and believe that I have an appropriate level of expertise to confirm that it is of an acceptable scientific standard.

# Response

**Published:** 2024-11-29

**Authors:** Didi Wahyudi

**Affiliations:** Telkom University, Bandung, West Java, Indonesia

Dear Reviewer,

 Thank you once again for your feedback.

# Reviewer report: Reviewer response for version 2

**DOI:** 10.5256/f1000research.174308.r338340

**Published:** 2024-11-08

**Authors:** Mohammed Zahedul Islam Nizami

**Affiliations:** 1 The Forsyth Institute, Harvard University, Cambridge, MA, USA

Thanks for your effort. It's much better now.

Is the rationale for developing the new method (or application) clearly explained?

Partly

Is the description of the method technically sound?

Partly

Are the conclusions about the method and its performance adequately supported by the findings presented in the article?

Partly

If any results are presented, are all the source data underlying the results available to ensure full reproducibility?

No

Are sufficient details provided to allow replication of the method development and its use by others?

Partly

Reviewer Expertise:

Cariology, preventive and restorative dentistry, dental biomaterials

I confirm that I have read this submission and believe that I have an appropriate level of expertise to confirm that it is of an acceptable scientific standard, however I have significant reservations, as outlined above.

# Response

**Published:** 2024-11-29

**Authors:** Didi Wahyudi

**Affiliations:** Telkom University, Bandung, West Java, Indonesia

Dear Reviewer,

 We have incorporated the limitations of the existing methods as suggested. We do not compare each existing method, not only because the focus of our writing does not lead in that direction, but also because they all utilize solid materials for obturation, thus sharing the same limitations based on the focus of our topic. This is a critical issue in endodontics due to the difficulty solid materials have in filling all the spaces within the root canal. This also serves as our rationale for considering a new method that uses gas (oxygen) as the obturation material. We have included this in the last paragraph of the "Current endodontic treatment" section, as per your suggestion.

 The consequence of placing limitations on existing methods in the "Current endodontic treatment" section makes the "three factors in endodontic treatment," which includes "obturation," better suited to also be included in the "Current endodontic treatment" section.

 We do not endorse any specific technique for "cleaning and shaping" or "disinfection" that must be employed prior to performing trepanation; therefore, we do not consider it necessary to include this in the clinical recommendations. This allows each dentist the flexibility to choose any technique as long as it effectively cleans and disinfects the root canal adequately before trepanation is performed.

 Clinical and radiological images, as you requested, are available in the Case Report that we cited. We hope that readers/clinicians have gained a clearer understanding of one example of a clinical case.

 Thank you once again for your feedback.

# Reviewer report: Reviewer response for version 1

**DOI:** 10.5256/f1000research.145403.r171942

**Published:** 2024-09-11

**Authors:** Rachmi Fanani Hakim, Fakhrurrazi Fakhrurrazi

**Affiliations:** 1 Department of Oral Biology, Faculty of Dentistry, University of Syiah Kuala, Banda Aceh, Indonesia; 2 Department of Oral and Maxillofacial Surgery, Faculty of Dentistry, Syiah Kuala university, Banda Aceh, Aceh, Indonesia

1. The strength of this article is that it offers a new method, but the weakness of writing a new method is that it lacks valid and up-to-date reference support and describes the problem less clearly.
2. The introduction of this article should discuss the current state of endodontic biomaterials, describing theoretical, experimental or observational, so that it is clear why it is necessary to review a new system in root canal treatment as the purpose of this article.
3. use the latest literature to explain concepts and trends in current endodontic care: Current Concepts and Trends in Endodontic Practice: An Update for Primary Dental Care (Blum I.et.al.2024 [Ref 2])
4. convey what are the advantages and disadvantages of current endodontic treatment
5. in the introduction it links endodontic treatment with covid, but in the section "Trepanation: bringing back oxygen circulation to a nonvital tooth" it explains that However, multiple-visit root canal treatment is not preferable for patients, not only during the COVID-19 pandemic. If you want to link it to COVID, it is better to convey it with clear literature, so as not to confuse the reader.
6. This sentence in the article must include clear, based and up-to-date references. ”Because the infection of the root canal of nonvital teeth tends to happen in an anaerobic condition, dental practitioners can consider preventing the root canal microenvironment from being oxygen-free. At some point, creating ventilation to avoid the absence of oxygen might look similar to trepanation. The size of the oxygen molecule is smaller than the diameter of dentinal tubules, making it able to fill the entire space available within the tooth. Oxygen can inhibit the growth of anaerobes and prevent facultative anaerobes from becoming more infectious. Using oxygen as an antimicrobial agent that fills the root canal is expected to shorten the working time of a dentist performing root canal treatments. It can replace conventional obturation after proper cleaning and shaping.”
7. This sentence in the article must include clear, based and up-to-date references. “In inactive caries, saliva certainly has no significant obstacle to contacting the tooth surface because no filling or anything is covering, but the bacteria are still not easy to progress. Suppose both secondary caries and inactive caries are exposed to saliva. In that case, the difference is that in inactive caries, the surface is more open and easier to clean to avoid debris adhesion. Inactive caries that do not develop for years is one reason to expect oxygen circulation can prevent infection in the root canal that priorly has been cleaned from various obstruction and contamination.”
8. This sentence in the article must include clear, based and up-to-date references. “Dental trepanation has been known by some, if not many, dental practitioners in Indonesia. It is also believed that the method still exists until recently. If this conventional method of dental trepanation - done without being followed by any modern technique of root canal preparation - was once considered beneficial for nonvital teeth, we hypothesize that a thoroughly cleaned, disinfected, and completely dried root canal, sealed with a specific restoration that uses an oxygen-permeable membrane, will give a better result. The membrane will let oxygen in while fluid, debris, and microorganisms cannot enter the root canal. The oxygen that keeps circulating to the entire root canal system and dentinal tubules will act as an antimicrobial agent that is constantly renewed to prevent the growth of pathogens inside the tooth. The novel membranous restoration system described below (please see Figures 1 – 4) is expected to decrease the working time in endodontic treatment, as it can replace the time-consuming conventional obturation techniques. Any conventional sealer for obturation, including those that use nanoparticles, and any established technique, cannot give a perfect seal in the root canal system. Naturally, oxygen can flow automatically and fill any space it can infiltrate. Using oxygen as obturation biomaterials will hopefully be easier and more time-saving than all current obturation techniques that use solid substances. Before applying this restoration design, proper root canal preparation, including debridement and disinfection, should be done first.”
9. This sentence in the article must include clear, based and up-to-date references. The oxygen-permeable membrane placed at the orifice of the duct is used to prevent any fluid, debris, and microorganisms from entering the root canal while letting oxygen go through. The membrane must also be of clinically safe material, durable to be placed in the mouth, and can be firmly attached to the selected restoration material. It should be noticed that the membrane must not be placed when the root canal is not clean and dry yet as the membrane cannot be passed by any fluid, including pus and irrigation solutions. In this case, it might be even better to use a membrane that can meet the following two criteria: it can let the gas flow from both sides of the membrane (two-way), and it can also let the fluid go through only from one side of the membrane (one-way)

Is the rationale for developing the new method (or application) clearly explained?

Partly

Is the description of the method technically sound?

Partly

Are the conclusions about the method and its performance adequately supported by the findings presented in the article?

Partly

If any results are presented, are all the source data underlying the results available to ensure full reproducibility?

No

Are sufficient details provided to allow replication of the method development and its use by others?

Partly

Reviewer Expertise:

bio enginering, stem cells research< natural product research, wound healing

We confirm that we have read this submission and believe that we have an appropriate level of expertise to confirm that it is of an acceptable scientific standard, however we have significant reservations, as outlined above.

## References

[1] : Pseudohypoparathyroidism with normal serum calcium level. *Am J Dis Child* .1975;129(9) : 10.1001/archpedi.1975.02120460072017 1092-5 10.1001/archpedi.1975.02120460072017 171942

[2] : Current Concepts and Trends in Endodontic Practice: An Update for Primary Dental Care. *Primary Dental Journal* .2020;9(4) : 10.1177/2050168420963336 10.1177/2050168420963336 33225853

# Reviewer report: Reviewer response for version 1

**DOI:** 10.5256/f1000research.145403.r237350

**Published:** 2024-05-25

**Authors:** Mohammed Zahedul Islam Nizami

**Affiliations:** 1 The Forsyth Institute, Harvard University, Cambridge, MA, USA

The aim of this study is good. Clinical trials should be shown to introduce clinical methodology. Please present at least one clinical case example. 

 In the “Current endodontic treatment” section, please compare and contrast the existing methods, their limitations, and the rationale of the proposed methodology.

 “three factors in endodontic treatment” should be discussed under the method section with a consistent clinical flow of the methodology with a flow diagram. 

 The proposed methodology should be under the method section using a subtitle. A schematic illustration following the clinical/radiological image of the proposed method should be presented to the reader/clinician to make it clearer.

Is the rationale for developing the new method (or application) clearly explained?

Partly

Is the description of the method technically sound?

Partly

Are the conclusions about the method and its performance adequately supported by the findings presented in the article?

Partly

If any results are presented, are all the source data underlying the results available to ensure full reproducibility?

No

Are sufficient details provided to allow replication of the method development and its use by others?

Partly

Reviewer Expertise:

Cariology, preventive and restorative dentistry, dental biomaterials

I confirm that I have read this submission and believe that I have an appropriate level of expertise to state that I do not consider it to be of an acceptable scientific standard, for reasons outlined above.

# Response

**Published:** 2024-11-01

**Authors:** Didi Wahyudi

**Affiliations:** Telkom University, Bandung, West Java, Indonesia

Thank you for the comments you have provided. We have included a case report on trepanation as you suggested. We agree that a clinical case example can help readers/clinicians, especially those who are not familiar with trepanation, to better visualize the concept. Although it took us some time to present this example, your suggestion was invaluable.

 We kindly ask you to recognize that the writing we have presented at this time does not delve into the specific advantages and disadvantages associated with some existing methods used in endodontic treatment. It is important to note that while our current work does not cover these aspects, there is potential for further research to be conducted in the future that could involve a detailed comparison of the different methods available.

 Once again, we greatly appreciate your suggestions regarding the clinical case example. This is what has caused us to take a long time to respond, as there has not yet been a paper that provides a sufficiently clear depiction of trepanation.

# Reviewer report: Reviewer response for version 1

**DOI:** 10.5256/f1000research.145403.r266227

**Published:** 2024-05-09

**Authors:** Tanay V Chaubal

**Affiliations:** 1 Periodontics, The University of Oklahoma Health Sciences Center College of Dentistry, OKLAHOMA CITY, Oklahoma, USA

I congratulate the authors in developing a novel membranous dental restoration system in endodontic management. The authors are aiming to change the environment inside the root canal system thereby making it more aerobic and unfavorable for the anaerobic bacteria like *E.faecalis* as it is the main culprit responsible for root canal failures. 

 However, there are a few shortcomings which need to be addressed. Firstly, have the authors carried out any in-vitro tests to study their hypothesis? Specifically microbiological tests to support their claim of reducing or eliminating the root canal microbiota. Secondly, the authors have to elaborate the limitations or shortcomings of their proposed system and at the same time provide future research or protocols to be followed for making a reduction in the incidence of root canal failure.

Is the rationale for developing the new method (or application) clearly explained?

Yes

Is the description of the method technically sound?

Yes

Are the conclusions about the method and its performance adequately supported by the findings presented in the article?

Partly

If any results are presented, are all the source data underlying the results available to ensure full reproducibility?

Partly

Are sufficient details provided to allow replication of the method development and its use by others?

Yes

Reviewer Expertise:

Periodontics, Oral Implantology, Systematic reviews and meta-analysis, Bone regeneration, Nanoparticles.

I confirm that I have read this submission and believe that I have an appropriate level of expertise to confirm that it is of an acceptable scientific standard, however I have significant reservations, as outlined above.

# Response

**Published:** 2024-11-01

**Authors:** Didi Wahyudi

**Affiliations:** Telkom University, Bandung, West Java, Indonesia

Thank you for the congratulatory messages; we truly appreciate them, though we recognize that our journey to further explore trepanation is still lengthy.

 Currently, we are striving to present facts about trepanation, a procedure used in Indonesia, for which no detailed paper exists. In fact, a government-owned dental hospital located in Bandung, Indonesia, lists "trepanasi," the Indonesian term for trepanation, as a service in both its pediatric dentistry (please see https://rskgm.bandung.go.id/klinik-pedodonti/ ) and endodontics (please see https://rskgm.bandung.go.id/klinik-konservasi-gigi/ ) departments.

 Our manuscript is an introductory text that serves as a starting point for a discussion on the topic of trepanation. This particular subject appears to have never been formally addressed in a comprehensive manner, even though it has been utilized by some, if not many, dental professionals in Indonesia. This writing is an initial effort that emphasizes a fundamental discussion regarding trepanation Please understand that the type of research that presents a hypothesis accompanied by logical analysis, supported by narratives reinforced with existing facts/cases and literature, is indeed acceptable for publication by many journals, including f1000research. This is to expedite the sharing of knowledge and existing phenomena. Further research, including those you have suggested, is hoped to be conducted in the future, either by us or by others who read this writing.

 However, we have included a discussion of a case report regarding trepanation, and we hope this can provide a clearer picture of trepanation. We also outline the limitations of the trepanation procedure mentioned in the case report and potential developments to address them, as you suggested. Thank you once again.
