# Supplementary material for: Oxygen as obturation biomaterial in endodontic treatment: development of novel membranous dental restoration system
Source: F1000Res. 2024 Nov 6;12:380. Originally published 2023 Apr 11. [Version 2] doi: 10.12688/f1000research.132479.2 (PMC11584453; doi:10.12688/f1000research.132479.2)
Supplement: Peer review history

# Response

**Published:** 2024-11-01

**Authors:** Didi Wahyudi

**Affiliations:** Dental Cooperation Indonesia, Bandung, Indonesia

Thank you for the comments you have provided. We have included a case report on trepanation as you suggested. We agree that a clinical case example can help readers/clinicians, especially those who are not familiar with trepanation, to better visualize the concept. Although it took us some time to present this example, your suggestion was invaluable.

 We kindly ask you to recognize that the writing we have presented at this time does not delve into the specific advantages and disadvantages associated with some existing methods used in endodontic treatment. It is important to note that while our current work does not cover these aspects, there is potential for further research to be conducted in the future that could involve a detailed comparison of the different methods available.

 Once again, we greatly appreciate your suggestions regarding the clinical case example. This is what has caused us to take a long time to respond, as there has not yet been a paper that provides a sufficiently clear depiction of trepanation.

# Response

**Published:** 2024-11-01

**Authors:** Didi Wahyudi

**Affiliations:** Dental Cooperation Indonesia, Bandung, Indonesia

Thank you for the congratulatory messages; we truly appreciate them, though we recognize that our journey to further explore trepanation is still lengthy.

 Currently, we are striving to present facts about trepanation, a procedure used in Indonesia, for which no detailed paper exists. In fact, a government-owned dental hospital located in Bandung, Indonesia, lists "trepanasi," the Indonesian term for trepanation, as a service in both its pediatric dentistry (please see https://rskgm.bandung.go.id/klinik-pedodonti/ ) and endodontics (please see https://rskgm.bandung.go.id/klinik-konservasi-gigi/ ) departments.

 Our manuscript is an introductory text that serves as a starting point for a discussion on the topic of trepanation. This particular subject appears to have never been formally addressed in a comprehensive manner, even though it has been utilized by some, if not many, dental professionals in Indonesia. This writing is an initial effort that emphasizes a fundamental discussion regarding trepanation Please understand that the type of research that presents a hypothesis accompanied by logical analysis, supported by narratives reinforced with existing facts/cases and literature, is indeed acceptable for publication by many journals, including f1000research. This is to expedite the sharing of knowledge and existing phenomena. Further research, including those you have suggested, is hoped to be conducted in the future, either by us or by others who read this writing.

 However, we have included a discussion of a case report regarding trepanation, and we hope this can provide a clearer picture of trepanation. We also outline the limitations of the trepanation procedure mentioned in the case report and potential developments to address them, as you suggested. Thank you once again.
